# Improvement of late gadolinium enhancement image quality using a deep learning–based reconstruction algorithm and its influence on myocardial scar quantification

**DOI:** 10.1007/s00330-020-07461-w

**Published:** 2020-11-21

**Authors:** Nikki van der Velde, H. Carlijne Hassing, Brendan J. Bakker, Piotr A. Wielopolski, R. Marc Lebel, Martin A. Janich, Isabella Kardys, Ricardo P. J. Budde, Alexander Hirsch

**Affiliations:** 1 Department of Cardiology, Erasmus MC, University Medical Center Rotterdam, Dr. Molewaterplein 40, 3015 GD Rotterdam, The Netherlands; 2 Department of Radiology and Nuclear Medicine, Erasmus MC, University Medical Center Rotterdam, Rotterdam, The Netherlands; 3 GE Healthcare, Calgary, Canada; 4 GE Healthcare, Munich, Germany

**Keywords:** Deep learning, Magnetic resonance imaging, Fibrosis, Heart

## Abstract

**Objectives:**

The aim of this study was to assess the effect of a deep learning (DL)–based reconstruction algorithm on late gadolinium enhancement (LGE) image quality and to evaluate its influence on scar quantification.

**Methods:**

Sixty patients (46 ± 17 years, 50% male) with suspected or known cardiomyopathy underwent CMR. Short-axis LGE images were reconstructed using the conventional reconstruction and a DL network (DLRecon) with tunable noise reduction (NR) levels from 0 to 100%. Image quality of standard LGE images and DLRecon images with 75% NR was scored using a 5-point scale (poor to excellent). In 30 patients with LGE, scar size was quantified using thresholding techniques with different standard deviations (SD) above remote myocardium, and using full width at half maximum (FWHM) technique in images with varying NR levels.

**Results:**

DLRecon images were of higher quality than standard LGE images (subjective quality score 3.3 ± 0.5 vs. 3.6 ± 0.7, *p* < 0.001). Scar size increased with increasing NR levels using the SD methods. With 100% NR level, scar size increased 36%, 87%, and 138% using 2SD, 4SD, and 6SD quantification method, respectively, compared to standard LGE images (all *p* values < 0.001). However, with the FWHM method, no differences in scar size were found (*p* = 0.06).

**Conclusions:**

LGE image quality improved significantly using a DL-based reconstruction algorithm. However, this algorithm has an important impact on scar quantification depending on which quantification technique is used. The FWHM method is preferred because of its independency of NR. Clinicians should be aware of this impact on scar quantification, as DL-based reconstruction algorithms are being used.

**Key Points:**

*• The image quality based on (subjective) visual assessment and image sharpness of late gadolinium enhancement images improved significantly using a deep learning–based reconstruction algorithm that aims to reconstruct high signal-to-noise images using a denoising technique.*

*• Special care should be taken when scar size is quantified using thresholding techniques with different standard deviations above remote myocardium because of the large impact of these advanced image enhancement algorithms.*

*• The full width at half maximum method is recommended to quantify scar size when deep learning algorithms based on noise reduction are used, as this method is the least sensitive to the level of noise and showed the best agreement with visual late gadolinium enhancement assessment.*

**Supplementary Information:**

The online version contains supplementary material available at 10.1007/s00330-020-07461-w.

## Introduction

The presence of myocardial fibrosis is a common finding in patients with both ischemic cardiomyopathy (ICM) and non-ischemic cardiomyopathy (NICM) [1, 2]. Cardiovascular magnetic resonance imaging (CMR) is the gold standard technique to visualize myocardial fibrosis by late gadolinium enhancement (LGE) imaging [3–6]. Determination of the presence and extent of LGE is of clinical importance as research has shown its prognostic value in the prediction of adverse cardiac events, including ventricular arrhythmias and sudden cardiac death [7–9]. In addition to visual assessment for LGE analysis, there are several threshold-based techniques to quantify the amount of myocardial fibrosis [10]. These threshold-based techniques are based on differences in signal intensity (SI) between fibrotic myocardium and normal remote myocardium [5]. In one of the techniques, the amount of hyperenhancement is determined by using different SI threshold values such as 2, 4, or 6 standard deviations (SD) above remote, normal myocardium. Another quantification technique known as full width at half maximum (FWHM) assumes a threshold value of half the maximum signal within the scar. The preference for a quantification technique differs per cardiac disease: the FWHM is frequently used for ICM and the SD threshold technique often used for NICM [3, 5, 11].

Despite these semi-automatic techniques, large variations in the extent of LGE between each quantification method still remain, due to the variability in noise, resolution, and intensity level of the LGE images and in patients (e.g., distribution pattern and extent of myocardial scar) [3–5, 12, 13]. By tackling one or more of these variabilities, the diagnostic accuracy of CMR images may improve. This can be achieved by obtaining images with high-resolution and high signal-to-noise ratio (SNR). However, with standard breath-hold imaging frequently used in CMR, SNR cannot be increased by increasing the number of averages (NEX) due to limited time [14]. Recently, there has been interest in the development and application of deep learning (DL) to reconstruct, enhance, or analyze medical images [4, 15–17]. Here, we investigated a new DL reconstruction algorithm (DLRecon) that aims to reconstruct high SNR images while preserving image detail using a denoising technique. The objective of this study was to establish the effect of this DL-based magnetic resonance imaging (MRI) reconstruction algorithm on LGE image quality and its influence on the quantification of myocardial scar in patients with ICM and NICM.

## Materials and methods

### Study population

For this single-center observational study, we screened patients who were referred for CMR at the Erasmus Medical Center, Rotterdam, the Netherlands, with proven or suspected ICM or NICM between March 2019 and April 2019. A total of 60 consecutive patients who underwent CMR including LGE images in the context of clinical care were included. No other in- or exclusion criteria were used. According to the institutional review board, this study did not meet the requirements of a study that is subject to the Medical Research Involving Human Subjects Acts.

### DL image reconstruction

The vendor-provided DLRecon prototype (GE Healthcare) uses a feed-forward deep convolutional neural network (CNN) that reconstructs images with higher SNR, reduced truncation artifacts, and higher spatial resolution [18]. This network architecture is a residual encoder, variants of which have been demonstrated as effective for highly related tasks, including image denoising, super resolution, and JPEG deblocking [19]. The CNN is integrated inside the standard reconstruction pipeline; accepts raw, unfiltered, complex-valued input images, and desired noise reduction (NR) level; and produces improved output images. The improved images have a noise variance that is reduced by the requested NR level, expressed as a percentage between 0 and 100% to accommodate user preference. NR 100% corresponds to removing of all the predicted noise from the image. The network also recognizes that Gibbs ringing occurs in the vicinity of sharp edges and achieves de-ringing to improve image sharpness. The result is an image with higher SNR and edge sharpness that is nearly free of truncation artifacts. The CNN contains over 4.4 million trainable parameters in over 10,000 kernels. It was trained with a supervised learning approach, using pairs of images representing near-perfect and conventional MRI images. The near-perfect training data consisted of high-resolution images with minimal ringing and very low noise levels. The conventional training data were synthesized from near-perfect images using established methods to create lower resolution versions with more truncation artifacts and with higher noise levels. A diverse set of training images spanning a broad range of image content were employed to enable generalizability of the CNN across all anatomies. Image augmentations, including rotations and flips, intensity gradients, phase manipulations, and additional Gaussian noise, were applied for added robustness, resulting in a training database of 4 million unique image/augmentation combinations. The network was trained with gradient backpropagation via the ADAM optimizer. This DLRecon method is developed for 2-dimensional (2D) anatomical sequences and is compatible with many standard sequences and options, including magnitude LGE short-axis (SA) views, long-axis views, fast single-shot acquisition, and phase-sensitive inversion recovery images.

### CMR patient protocol

CMR examinations were performed on a 1.5T whole body clinical MR system (SIGNA Artist, GE Healthcare) with a dedicated anterior array coil, electrocardiographic gating, and breath-hold techniques. The imaging protocol consisted of balanced steady-state free precession cine images and 2D LGE imaging. LGE imaging was performed 10–20 min after intravenous administration of a gadolinium-based contrast agent (0.15 to 0.2 mmol/kg; Gadovist, Bayer Healthcare), using a breath-held 2D-segmented inversion recovery gradient echo pulse sequence with magnitude reconstruction. LGE images were obtained in standard long-axis and SA views, with coverage from base to apex. Typical scan parameters were slice thickness 8 mm, interslice gap 2 mm, TR/TE 6.5/3.0 ms, flip angle 25°, ASSET 1.5, NEX 1, field of view 256–410 × 320–430 mm, acquired matrix 200 × 192, and reconstructed to a pixel size of 1.3–2.1 × 1.1–1.5 mm. If necessary, the preset inversion time was adjusted to null normal myocardium.

LGE images were reconstructed multiple times from the same source data: once using the vendor standard reconstruction, then again using the vendor-supplied DLRecon prototype. For this study, LGE images were reconstructed with a NR level of 25%, 50%, 75%, and 100%.

### Phantom scan protocol

Static phantom scans were performed, with the application of tunable NR levels of 25%, 50%, and 75%, to demonstrate the relationship between different NR levels and improvement in SNR. Scans were performed on the same 1.5T MR system with a doped static phantom and 32-channel anterior array coil. A 2D-segmented inversion recovery gradient echo pulse sequence with magnitude reconstruction was used, with an inversion preparation time of 110 ms, emulated heart rate at 100 bpm, and otherwise identical parameters as in the patient study. To quantify NR levels, the data acquisition was repeated with multiple averages from 1 to 16 NEX. Mean SI and SD were measured by taking the average of three circularly drawn regions of interest (ROI) outside the phantom, and SNR was calculated by the quotient of SI and SD, according to IEC standards [20].

### CMR analysis

**Fig. 1:**
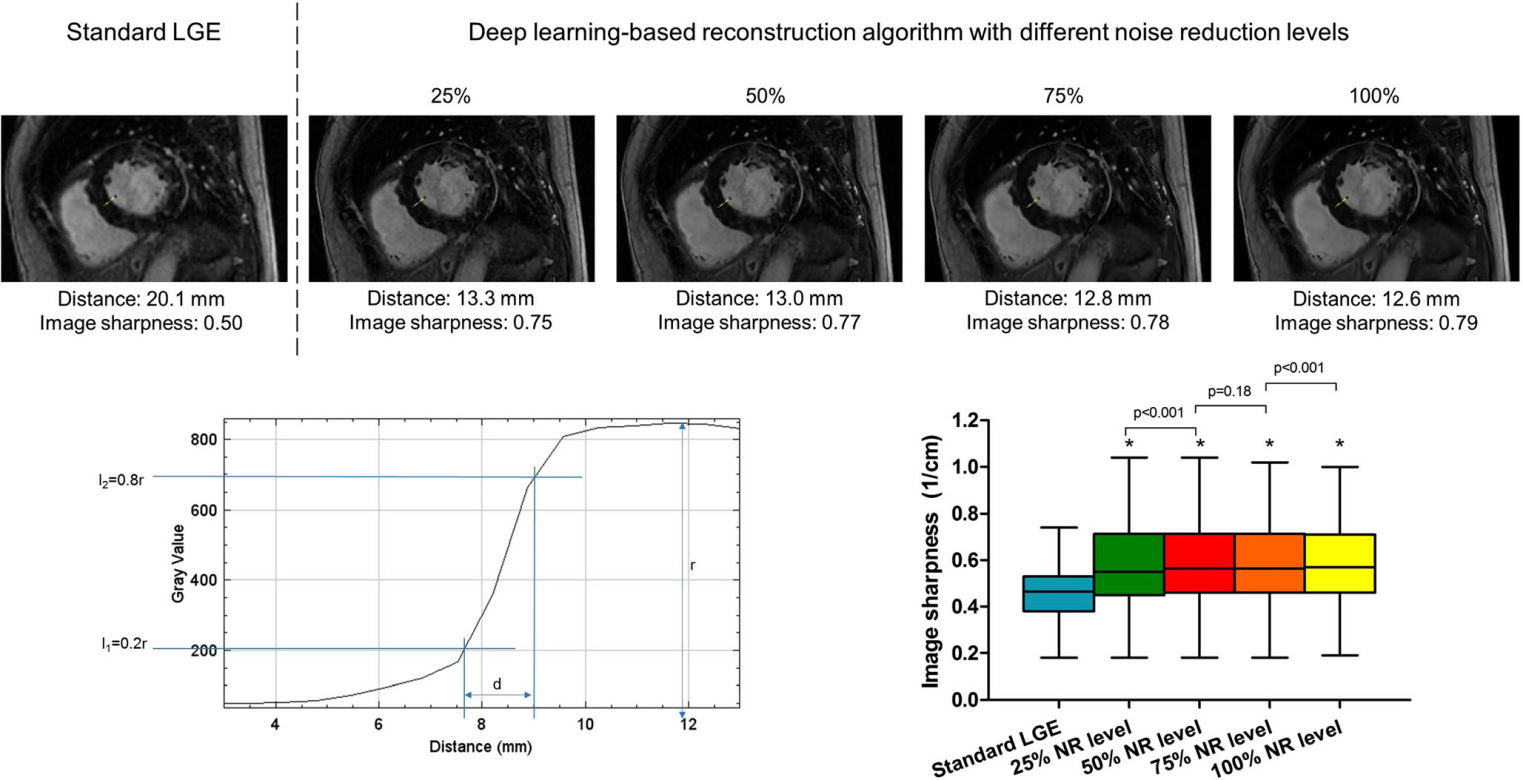
Assessment of image sharpness. Example of an endocardial border sharpness measurement in both standard late gadolinium enhancement (LGE) images and DLRecon images with 25%, 50%, 75%, and 100% noise reduction levels (top row). For each profile (example bottom left), image sharpness was calculated by taking the inverse of the distance (d) between 20 and 80% of the total intensity range (*r*) of the profile. Image sharpness (1/cm) of all 60 patients is depicted in the box plots (bottom right). The deep learning–based reconstruction algorithm resulted in a significantly increased image sharpness. *Significantly different compared to standard LGE; *p* < 0.001)

All 2D SA LGE images were analyzed to determine the effect of DLRecon on image quality and LGE quantification. Image quality and myocardial nulling were assessed on standard LGE images and DLRecon images with 75% NR level. These assessments were performed blinded and independently in all 60 patients by two experienced imaging cardiologists (A.H. and C.H.) and one experienced researcher (N.vdV.) using a 5-point Likert scale (1 = poor, 2 = fair, 3 = good, 4 = very good, 5 = excellent). Moreover, the visibility of artifacts (1 = severe image artifacts, 2 = moderate to severe image artifacts, 3 = moderate image artifacts, 4 = mild image artifacts, 5 = no image artifacts) and the presence of hyperenhancement (yes or no) was scored. Also, image sharpness, as a more objective method for image quality, was calculated in standard LGE images as well as in the DLRecon images with NR levels of 25%, 50%, 75%, and 100% using open-source software ImageJ (National Institutes of Health). In each mid ventricular SA slice with the least myocardial trabeculations, a single profile was selected along the septal myocardium (Fig. 1). This profile was copied between the standard LGE images and the DLRecon images. Sharpness was calculated by taking the inverse of the distance between 20 and 80% of the pixel intensity range of the profile [21].

In addition, SNR and contrast-to-noise ratio (CNR) were determined, as criteria for image quality, in patients with hyperenhancement (*n* = 30). These measurements were performed by drawing ROIs in remote myocardium, hyperenhanced myocardium, and air signal outside the patient. SNR of the scar was measured as mean SI of the hyperenhanced myocardium divided by SD of the air signal outside the patient. CNR between scar and remote myocardium was calculated as (mean SI of hyperenhanced myocardium − mean SI of remote myocardium)/(1.5 × SD of the air signal outside the patient) [5].

In the same subset of patients with hyperenhancement, additional analyses were performed with regard to LGE analysis. Epicardial and endocardial contours (excluding papillary muscles) of the left ventricle (LV) were manually drawn on each slice of the SA LGE images, using dedicated software (QMass software version 8.1, Medis Medical Imaging Systems bv). Subsequently, two regions of interest ROIs, one in hyperenhanced myocardium and one in normal remote myocardium, were drawn automatically in the SA slice where hyperenhancement was visually most pronounced. After visual inspection and manual adjustments if necessary, the contours were copied between standard LGE images and DLRecon images with NR levels of 25%, 50%, 75%, and 100%. Thereafter, hyperenhanced myocardium was automatically quantified as percentage of the LV using different quantification techniques: the thresholding technique with 2SD, 4SD, and 6SD above remote myocardium and the FWHM method [10]. For the manual technique, hyperenhanced myocardium was drawn by visual assessment of each SA slice.

### Statistical analysis

All continuous data were tested for normality before analysis using the Kolmogorov-Smirnov test or Shapiro-Wilk test, depending on the number of patients, and were expressed as mean ± SD or median (interquartile range (IQR)), as appropriate. Categorical variables were presented as number (%). Wilcoxon signed-rank tests were used for the comparison of differences in image quality, severity of artifacts, myocardial nulling, and for the comparison of extent of LGE between different quantification techniques. Intraclass correlations (ICC) were used to evaluate the agreement between observers and were interpreted as follows: < 0.2 = poor, 0.21–0.40 = fair, 0.41–0.60 = moderate, 0.61–0.80 = good, and 0.81–1.00 = excellent. Paired *t* tests were used for the comparison of the image sharpness. For each quantification method, the amount of LGE as percentage of the LV between the standard LGE images and the images with different NR levels was compared by performing the Friedman’s test. If a significant difference was found, a Wilcoxon signed-rank test with Bonferroni correction was performed to determine the exact difference between the standard LGE images and images with varying NR levels. All analyses were two-tailed; after correction for multiple testing, a *p* < 0.0125 was considered as statistically significant. Statistical analyses were performed using SPSS (version 21, IBM SPSS Statistics, IBM corporation).

## Results

### Phantom scans

**Fig. 2:**
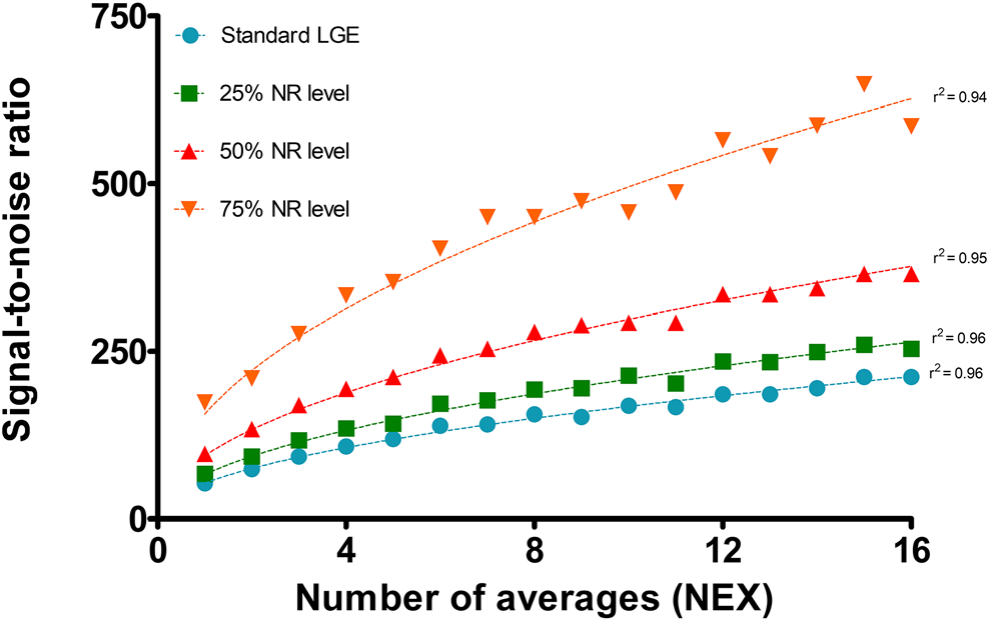
Relationship between signal-to-noise ratio and the number of averages stratified according to different DLRecon noise reduction levels. Square root relationship between signal-to-noise ratio and number of averages in standard late gadolinium enhancement (LGE) images and in deep-learning-reconstructed (DLRecon) images with noise reduction (NR) levels of 25%, 50%, and 75%

Figure 2 illustrates the relationship between SNR and NEX without and with DLRecon with NR levels of 25%, 50%, and 75%. As depicted in Fig. 2, here is a square root relationship between SNR and NEX. The SNR of an image with 50% or 75% NR level (and NEX 1) is comparable with an image with 4 NEX or 16 NEX without NR, respectively. Consequently, with a 50% or 75% NR factor, the SNR increased by a factor 1.8 and 3.0 compared to the standard LGE images.

### Image quality of patient scans

Baseline patient and CMR characteristics are presented in Table 1. Mean age of the study group was 46 ± 17 years and 50% were males.

**Table 1:** Baseline patient and CMR characteristics

	All patients (*n* = 60)
Demographic characteristics	
Age (years)	46 ± 17
Male gender	30 (50%)
Height (in cm)	175 ± 10
Weight (in kg)	83 ± 17
BSA (in m^2^)	2.0 ± 0.2
CMR diagnosis	
Normal	20 (33%)
Hypertrophic cardiomyopathy	13 (22%)
Dilated cardiomyopathy	7 (12%)
Other cardiomyopathy	11 (18%)
Myocardial infarction	8 (13%)
Myocardial ischemia without infarction	1 (2%)
CMR parameters	
LV end-diastolic volume (ml)	175 (146–199)
LV end-systolic volume (ml)	77 (61–96)
LV stroke volume (ml)	93 ± 22
LV ejection fraction (%)	56 (51–61)
Late gadolinium enhancement	Patients with LGE (*n* = 30)
Ischemic LGE distribution pattern	8 (27%)
Non-ischemic LGE distribution pattern	22 (73%)
Percentage LGE of LV (manual)	6 (5–11)

Continuous data presented as mean + SD or as median with IQR. Categorical data presented as number (%)

*CMR* cardiovascular magnetic resonance imaging, *LGE* late gadolinium enhancement, *LV* left ventricle

**Fig. 3:**
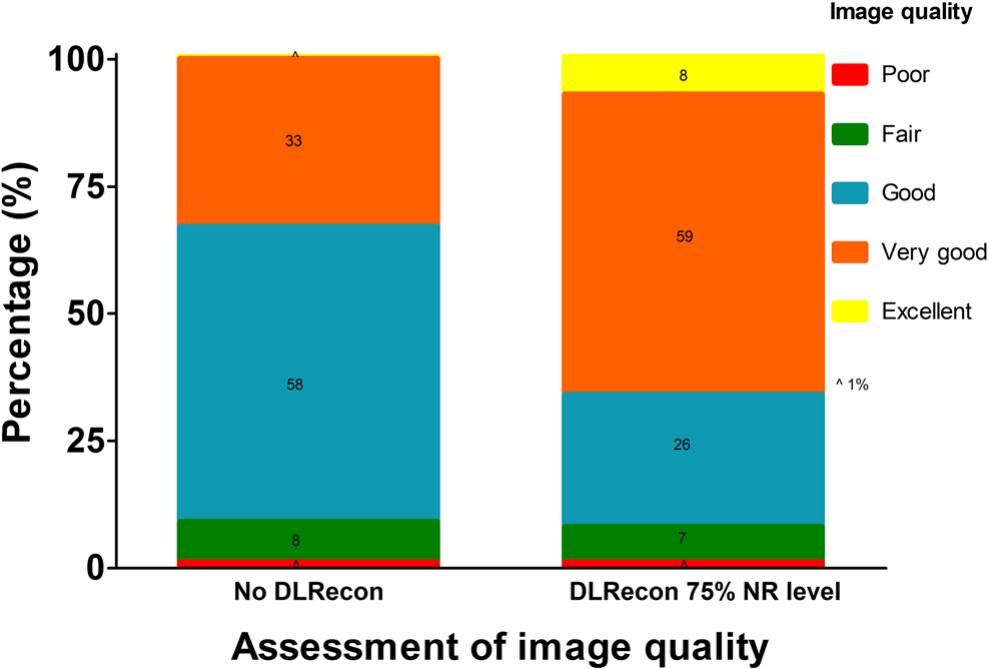
Assessment of image quality. Assessment of the image quality in percentages of both standard late gadolinium enhancement and deep learning–reconstructed (DLRecon) images with a 75% noise reduction (NR) level by three observers in all 60 consecutive patients

Assessment of image quality, artifacts, and myocardial nulling of both standard LGE and DLRecon images with 75% NR level was performed in all patients. Agreement between the three observers was moderate to good for image quality (ICC 0.52 for standard LGE images and 0.62 for DLRecon images with 75% NR level), moderate for visibility of artifacts (ICC 0.58 and 0.59, respectively), and fair to moderate for myocardial nulling (ICC 0.39 and 0.44, respectively). Image quality improved significantly by applying the DLRecon method, with an increase in mean image quality from 3.2 ± 0.5 to 3.7 ± 0.6 (*p* < 0.001, Fig. 3). Nevertheless, the visibility of artifacts, especially wrapping and ghosting artifacts, was emphasized when DLRecon was used (3.7 ± 0.5 vs. 3.4 ± 0.7, *p* < 0.001). Furthermore, in DLRecon images, no improvement in distinction between suppressed and hyperenhanced myocardium was achieved (3.3 ± 0.5 vs. 3.3 ± 0.6, *p* = 0.30). No difference in the presence and visual extent of LGE was found between standard and DLRecon images.

**Fig. 4:**
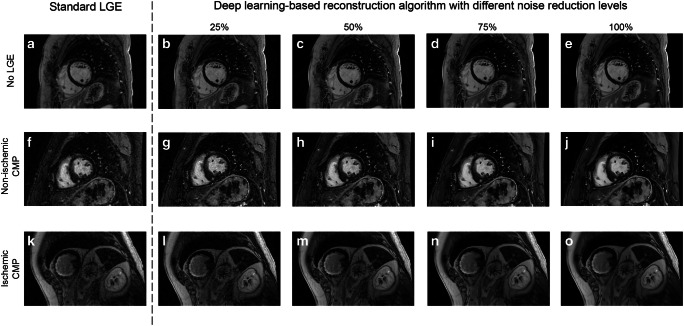
LGE image example with and without applying a deep learning–based reconstruction algorithm. Examples of late gadolinium enhancement (LGE) images with the standard and deep learning–based reconstruction algorithm using different noise reduction levels in a patient without hyperenhancement (images **a–e**), in a patient with hyperenhancement with a non-ischemic distribution pattern (images **f–j**), and in a patient with hyperenhancement due to ischemic cardiomyopathy (CMP) (images **k–o**). The new reconstruction algorithm resulted into a higher signal-to-noise ratio with increasing noise reduction levels, reduction in truncation artifacts, and sharper edges

In addition to a significant increase in image quality, the image sharpness between standard LGE images and DLRecon images with 25%, 50%, 75%, and 100% also increased significantly (0.46 ± 0.11 vs. 0.58 ± 0.18 vs. 0.59 ± 0.17 vs. 0.59 ± 0.17 1/cm, respectively, all *p* values < 0.001; Fig. 1). In patients with hyperenhancement (*n* = 30), both SNR_scar_ and CNR_scar-remote_ increased with 121% and 127%, respectively (both *p* < 0.001). Examples of LGE images with and without DLRecon are shown in Fig. 4.

### Myocardial scar size quantification

**Fig. 5:**
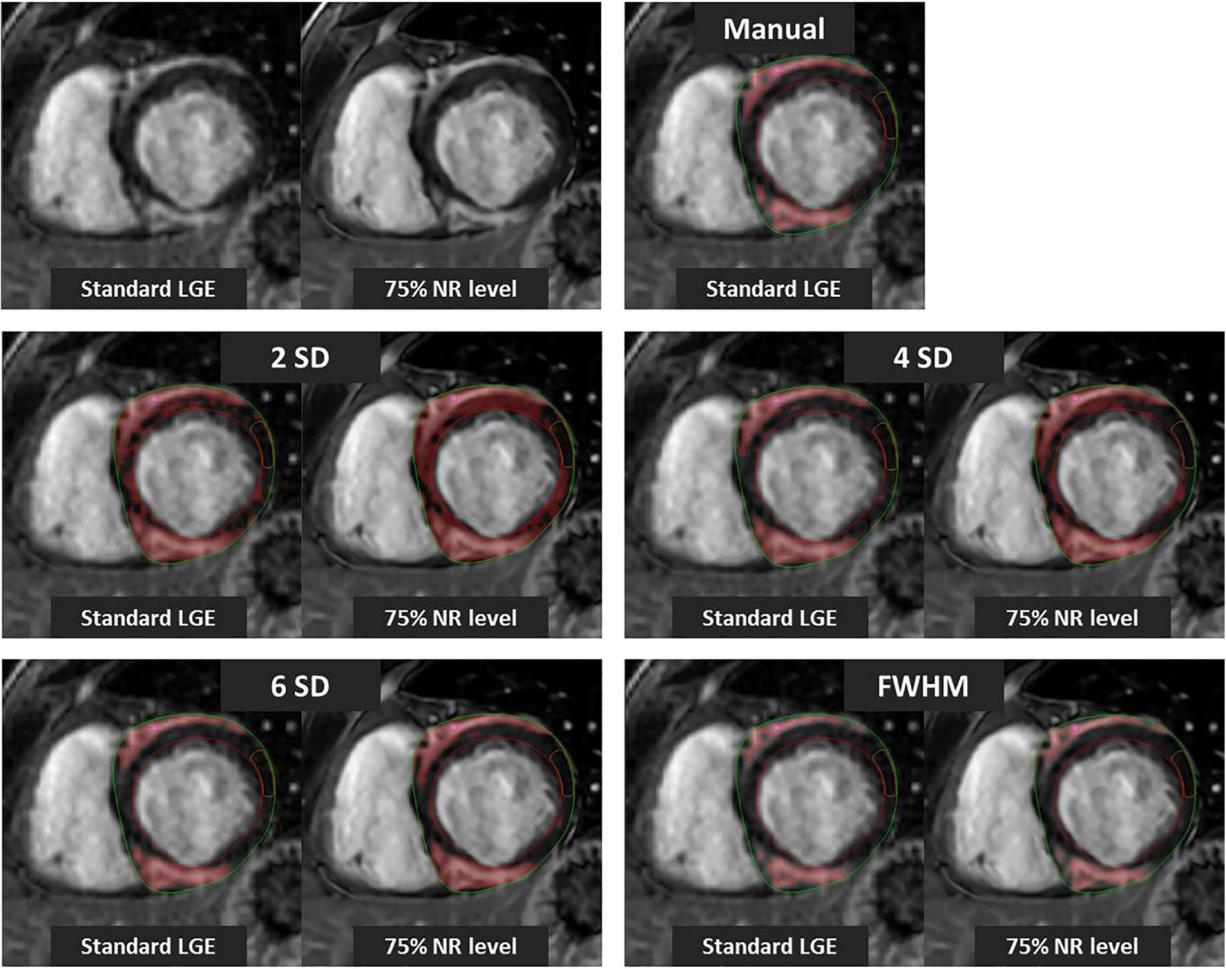
LGE quantification using different threshold-based techniques. Example of the different quantification methods in a standard short-axis (SA) late gadolinium enhancement (LGE) image and in the same SA reconstructed with a 75% noise reduction (NR) level in a patient with non-ischemic cardiomyopathy. The threshold technique by standard deviation (SD) led to an increase in hyperenhancement area in the LGE image reconstructed with 75% NR level compared to standard LGE image. There was no difference in the hyperenhanced area in the full width at half maximum (FWHM) method

**Fig. 6:**
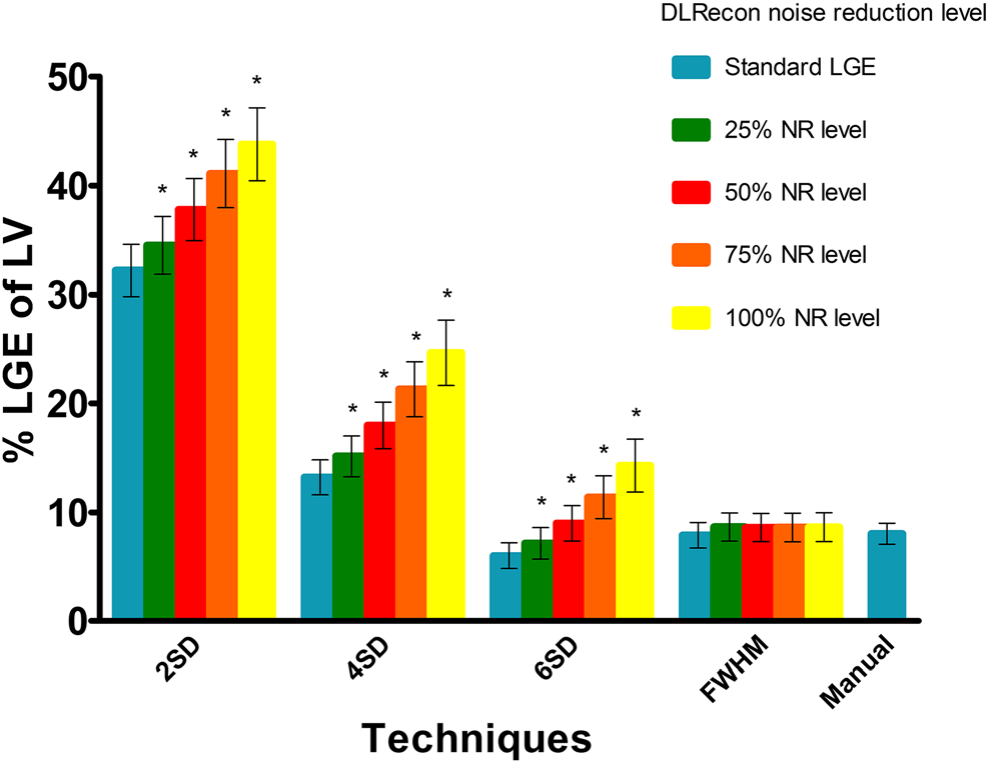
Scar size quantification. Quantification of hyperenhancement as percentage of left ventricle (LV) using different techniques: thresholding by standard deviation (SD) above remote myocardium, full width at half maximum (FWHM) and manual. Scar size was quantified in standard late gadolinium enhancement (LGE) images and in deep learning-reconstructed images (DLRecon) images stratified according to noise reduction (NR) level (from 25 to 100%). Data presented as mean with standard error of the mean. *Significantly different compared to standard LGE; *p* < 0.0125 considered as statistically significant

Hyperenhancement was observed in 30 patients; 73% had a non-ischemic distribution pattern. Figure 5 shows an example of the different quantification methods in a standard SA LGE image and in the same SA reconstructed with 75% NR level. There was a significant increase in percentage of hyperenhancement of the LV with incremental NR levels when the SD thresholding techniques were used (Fig. 6 and supplementary Table 1). The extent of hyperenhancement increased by 36%, 87%, and 138%, respectively, using 2SD, 4SD, and 6SD quantification methods between standard LGE images and images with 100% NR level (all *p* values < 0.001). This difference was not found using the FWHM method, where myocardial scar size was independent of NR levels (Friedman’s *p* = 0.06). The extent of hyperenhancement was 9% larger using images with 100% NR level compared to standard LGE images; however, this was not significant (*p* = 0.053). When the different quantification techniques with the application of DLRecon were compared to manual LGE quantification, the best agreement was found with the FWHM method (*p* = 0.49). The SIs and the different thresholds are depicted in supplementary Table 2.

The same results were found with regard to increase in extent of LGE between standard LGE and DLRecon images when patients were stratified according to ICM or NICM (supplementary Fig. 1). In ICM patients, manual measurement of scar size in standard LGE images was consistent with 6SD technique (*p* = 0.48) and the FWHM resulted in a smaller infarct size (*p* = 0.017). In NICM patients, the manual measurement was most comparable to the FWHM method in standard LGE images (*p* = 0.41).

## Discussion

In this study, we evaluated the impact of a DL-based reconstruction algorithm on LGE image quality and its effect on the quantification of myocardial fibrosis was assessed. Our main findings can be summarized as follows: (1) image quality by visual scoring improved significantly and consistently by applying DLRecon with a NR level of 75%; (2) in static phantom scans, DLRecon with NR levels of 50% and 75% increased SNR by a factor 1.8 and 3.0 compared to the standard LGE images; (3) both image sharpness and SNR and CNR increased significantly in clinical acquired images; and (4) DLRecon has a considerable impact on LGE extent using SD threshold techniques.

### Image quality and DLRecon

As it is known, image quality can be improved by increasing the NEX, providing high SNR images. However, this also increases scan duration. Therefore, several studies have recently investigated the influence of applying various DL-based noise and artifact reduction techniques on different types of sequences, for the purpose of retaining or improving MRI image quality and reducing scanning time within various radiological disciplines [14–17, 22, 23]. In addition to the comparison of different denoising methods, one of these studies also evaluated its effect on image quality between images with varying NEX values and DL-based-reconstructed images with diverse NR levels. It was shown that the reconstructed images with shorter acquisition time yielded equal or even better image quality than the images which were acquired with a higher NEX. In addition, small, precise anatomical structures remained clearly visible in the reconstructed images [14]. The same effect concerning SNR was achieved with the performed phantom scans in our study. This demonstrates that DL-based denoising techniques are effective in improving image quality, while a short acquisition time is retained. Two other studies demonstrated a retained or increased SNR when a DL-based NR technique was used, even while artifacts were also removed in one of the studies [17, 22]. The study of Hauptmann et al showed superior image quality and no statistically significant differences in functional ventricular measurements of undersampled real-time MRI images, compared to the reference standard, cardiac-gated, breath-hold techniques. These findings can strongly benefit patients, as image quality improves, while being able to reduce examination duration by omitting sequences with breath-holds. Our study confirmed the SNR findings with phantom scans, since SNR of our images improved by a factor 1.8 or 3.0 by applying DLRecon NR levels of 50% or 75%, respectively. However, ghosting and wrapping artifacts became more pronounced when LGE images were reconstructed with higher NR levels. Nevertheless, our overall image quality improved significantly by applying DLRecon.

### Effect of DLRecon on LGE extent

Our study confirmed, just like previous studies, that the extent of LGE in patients with ICM and NICM varied widely depending on the quantification technique that was used [3, 5, 24]. The use of a wrongly chosen quantification technique may lead to either under- or overestimation of the amount of myocardial fibrosis [3, 11].

Although research has been done to establish the most optimal LGE quantification method, no consensus has yet been reached [3, 5, 10, 12, 24–27]. In summary, these studies demonstrated that the most suitable quantification method differs per LGE distribution pattern, where the distribution pattern in patients with ICM generally corresponds to a particular coronary artery territory in contrast to less defined, diffuse, multifocal and patchy LGE areas in patients with NICM [12, 28]. Our study showed that LGE could be best quantified with the FWHM method as this method is most closely resembles the manual method. However, the SD method is still regularly used as quantification method, especially in patients with NICM [29–31].

It is already known that the use of denoising techniques can influence image quality and with that probably also the scar visualization on LGE images, but to our knowledge, the magnitude of this effect is not known [32]. Assessing the presence and accurate determination of the amount of myocardial fibrosis is of clinical importance, since multiple studies have shown that the presence and extent of LGE is of prognostic value in the prediction of all-cause mortality, cardiovascular mortality, ventricular arrhythmia, and SCD [7–9]. For example, studies have shown a relation between the extent of LGE and the risk of SCD in HCM [33–35]. Therefore, several clinicians consider the extent of fibrosis when recommending an implantable cardioverter-defibrillator for primary prevention, especially in those at intermediate risk of SCD. Our study showed that the amount of myocardial fibrosis almost doubled if LGE images were reconstructed with the highest NR level, depending on the used quantification method. A plausible explanation for this is that the SD-based method depends on the SI and SD of the signal of the ROI in the remote myocardium. Our data showed that the SD of the signal of the ROI in the remote myocardium is reduced due to NR with DLRecon, resulting in lower SI thresholds. This may lead to an increase in LGE extent and possible over- or mistreatment of patients. Clinicians should be aware of this risk. Besides, quantification of the extent of LGE using SD threshold technique is highly affected by the amount of noise/SNR; this can be influenced by a lot of other factors like static magnetic field strength, radiofrequency receive coil array, excitation flip angle, and receive bandwidth. However, the FWHM method does not depend on the SD of SI of the remote ROI but uses a threshold based on the SI within the remote and hyperenhanced area, making that technique less dependent on the different NR levels.

This study should function as a warning not to implement cutoffs from other studies based on SD threshold technique without careful consideration of these limitations. With regard to the DLRecon algorithm adapting the cutoffs for the extent of LGE to the NR level used is a possibility using the data provided in the manuscript. However, despite the possible disadvantage of FWHM method in especially NICM due to the diffuse fibrosis, we recommend using this method instead of SD threshold methods. The FWHM method is the least sensitive to the level of noise and showed the best agreement with visual late gadolinium enhancement assessment.

### Study limitations

Our study has several limitations: first, image quality assessment could not be assessed in a blinded way as it was clear for the reviewers which image was the standard LGE image and which image was reconstructed with 75% NR level. Secondly, this study was a single-center study with a relatively small sample size, especially the number of patients with ICM. There was a trend to an increase in the extent of hyperenhancement using the FWHM method with DLRecon over conventional reconstruction; however, this was not significant (9%, *p* = 0.053). The FWHM method may prove statistically different with additional subjects, but is likely to remain less sensitive than the standard deviation method. Finally, we did not investigate the possibility of altering scan parameters for improved image quality. Higher resolution protocols with adequate CNR or faster protocols with comparable resolution may be possible and further improve image quality.

## Conclusions

Myocardial LGE image quality improved significantly using a DL-based reconstruction algorithm. However, this NR algorithm has important impact on scar size quantification depending on which quantification technique was used. Clinicians and researchers should be aware of this large impact on scar quantification because advanced image reconstruction or other image enhancement algorithms are becoming more and more available and this can have major clinical consequences. Our study recommends the FWHM method with application of NR levels up to 100% when DL algorithms are used, as this method is the least sensitive to the level of noise and showed the best agreement with visual assessment of LGE.

## Supplementary information

ESM 1 (DOCX 179 kb)

## Abbreviations

2D: 2-Dimensional
CMR: Cardiovascular magnetic resonance imaging
CNR: Contrast-to-noise ratio
DL: Deep learning
DLRecon: Deep learning–based reconstruction algorithm
FWHM: Full width at half maximum
ICM: Ischemic cardiomyopathy
LGE: Late gadolinium enhancement
LV: Left ventricle
MRI: Magnetic resonance imaging
NEX: Number of averages
NICM: Non-ischemic cardiomyopathy
NR: Noise reduction
ROI: Region of interest
SA: Short-axis
SD: Standard deviation
SEM: Standard error of the mean
SI: Signal intensity
SNR: Signal-to-noise ratio
