# Supplementary material for: Improvement of late gadolinium enhancement image quality using a deep learning–based reconstruction algorithm and its influence on myocardial scar quantification
Source: Eur Radiol. 2020 Nov 21;31(6):3846–55. doi: 10.1007/s00330-020-07461-w (PMC8128730; doi:10.1007/s00330-020-07461-w)
Supplement: Supplementary file 1 — (DOCX 179 kb) [file 330_2020_7461_MOESM1_ESM.docx]

**Supplementary Table 1: Scar size quantification stratified according to noise reduction level (from 25-100%)**

|  | **Standard LGE** | **Deep learning based reconstruction algorithm with different**  **noise reduction levels** | | | |  |
| --- | --- | --- | --- | --- | --- | --- |
| **Technique** |  | **25%** | **50%** | **75%** | **100%** | **p-value** |
| **2SD** | 30.9  [22.2 – 42.5] | 35.0  [25.0 – 43.0] | 39.3  [27.7 – 46.7] | 41.3  [30.1 – 52.4] | 43.1  [31.6 – 58.2] | <0.001 |
| **4SD** | 11.7  [7.9 – 16.1] | 13.4  [9.0 – 17.5] | 14.5  [9.9 – 22.2] | 15.8  [11.0 – 27.4] | 17.1  [12.1 – 39.9] | <0.001 |
| **6SD** | 4.7  [2.2 – 7.0] | 5.5  [2.9 – 8.9] | 6.9  [3.8 – 11.0] | 8.1  [4.0 – 14.1] | 9.7  [5.2 – 21.5] | <0.001 |
| **FWHM** | 4.8  [3.5 – 11.9] | 5.1  [3.6 – 12.4] | 5.2  [3.7 – 12.4] | 5.3  [3.8 – 12.6] | 5.5  [3.9 – 12.5] | 0.06 |
| **Manual** | 6.1  [5.3 – 10.7] | - | - | - | - | - |

Data presented as median with IQR. P-values are from Friedman’s test.

Abbreviation: FWHM = Full width at half maximum, LGE = Late gadolinium enhancement, SD = Standard deviation

**Supplementary Table 2: Signal intensity with standard deviation of remote and hyperenhanced areas and the different thresholds in patients with LGE (n=30)**

|  | **ROI remote area** | | **ROI hyperenhancement** | | **Mean signal intensity threshold**  **2SD (au)** | **Mean signal intensity threshold**  **4SD (au)** | **Mean signal intensity threshold**  **6SD (au)** | **Mean signal intensity threshold FWHM (au)** |
| --- | --- | --- | --- | --- | --- | --- | --- | --- |
|  | **Mean signal intensity (au)** | **Mean SD of the  signal intensity (au)** | **Mean signal intensity  (au)** | **Mean SD of the  signal intensity (au)** |  |  |  |  |
| **Standard LGE** | 56 | 27 | 335 | 59 | >110 | >164 | >218 | >196 |
| **25% NR level** | 53 | 25 | 336 | 55 | >103 | >153 | >203 | >195 |
| **50% NR level** | 50 | 23 | 334 | 53 | >96 | >142 | >188 | >192 |
| **75% NR level** | 47 | 21 | 333 | 51 | >89 | >131 | >173 | >190 |
| **100% NR level** | 45 | 19 | 331 | 49 | >83 | >121 | >159 | >188 |

Abbreviations: FWHM = Full width half maximum, LGE = Late gadolinium enhancement, NR = Noise reduction, SD = Standard deviation


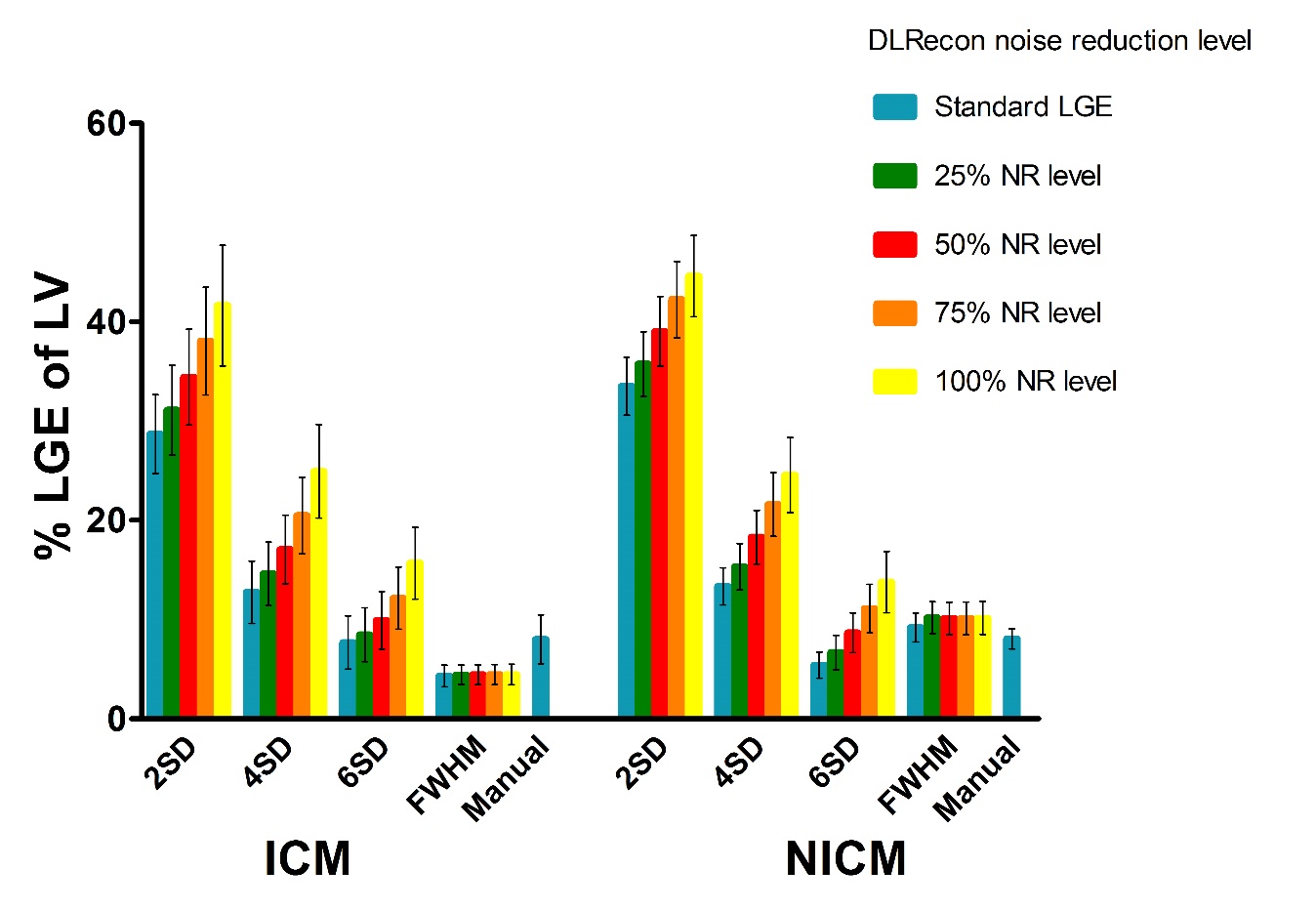


**Supplementary figure 1: Comparison of scar size as percentage of the LV between ischemic cardiomyopathy and non-ischemic cardiomyopathy using different quantification techniques**

Quantification of late gadolinium enhancement as percentage of left ventricle between ischemic cardiomyopathy and non-ischemic cardiomyopathy using different techniques: thresholding by standard deviation above remote myocardium, full width at half maximum and manual. Data presented as mean with standard error of the mean.

Abbreviation: DLRecon = Deep learning based reconstruction algorithm, FWHM = Full width at half maximum, ICM = Ischemic cardiomyopathy, LGE = Late gadolinium enhancement, LV = Left ventricle, NICM = Non-ischemic cardiomyopathy, SD = Standard deviation
